# The Treatment of Pneumonia

**Published:** 1893-03-25

**Authors:** 


### ST. MARY'S HOSPITAL.

### The Treatment of Pneumonia.

Pneumonia is an example of one of the many acute forms of disease in which it is almost impossible to lay down hard and fast rules with regard to treatment. The reason for this is due, in a large measure, to the great difference with respect to severity and type of the various epidemics of pneumonia, and to the varying powers of resistance shown by different individuals. It is also partly due to the fact that physicians are as yet by no means agreed as to the best mode of treatment of the disorder.

This article will deal with the general plan of treatment pursued at St. Mary's Hospital, and reference will be made, where necessary, to the more important modifications adopted by the different physicians. It will be convenient to discuss the treatment in relation to the different stages into which the course of the disease is divided in the ordinary text books on medicine.

These stages are three in number, viz.: (1) The first stage or stage of hyperæmia. (2) The second stage or stage of consolidation. (3) The third stage or stage of crisis and resolution.

*The Stage of Hyperæmia.*—The patient is confined to bed in a large and airy ward, the temperature of which is maintained at about 62 deg. to 65 deg. F. The temperature is taken every four hours in ordinary cases. Sponging with tepid water often gives great relief, and it might be done night and morning as a routine practice.

The diet consists of milk and beef-tea only, and the patients fed at short and regular intervals.

It is the usual custom at St. Mary's to commence the treatment by giving one to two grains of calomel with ten to thirty grains of pulv. jalap co., followed, if necessary, by a saline, by which means a free evacuation of the bowels is produced.

The local treatment will be first discussed, and it will be necessary to consider it in some detail.

Pain in the side—often a distressing symptom—may be treated with hot poultices frequently renewed, with turpentine stupes, or even by cold compresses. In robust patients the application of a few leeches (six to ten) generally gives immediate and lasting relief. Should leeches be contraindicated (as in weakly or anæmic individuals), the application of a blister may be tried, or the subcutaneous injection of morphia.

The affected side is enveloped in a linseed-meal poultice, changed every four hours, or turpentine stupes applied from time to time. It is of importance that the movements of the unaffected side should not be hampered in any way. When the local applications are discontinued the side is protected by a few layers of cotton wool.

Of late years Dr. Lees has used the ice-bag in the treatment of pneumonia, and as the result of his experience he now warmly advocates its employment as a local application. He has now come to the conclusion that not only does the ice of the ice-bag relieve the pain, and greatly reduce the temperature, but also that it tends to check local inflammation of the lung. It is necessary to add a few words with regard to the method of employment of this remedy, and also with respect to the precautions to be adopted while using it. The bag—in an emergency an ordinary sponge-bag will suffice—is to be filled loosely with small pieces of ice, and the mouth securely tied—a soft towel placed round the bag will absorb any liquid that may escape from it. The bag is then to be placed in contact with as large a surface of the affected side as possible, and should the patient be restless, may be kept in position by a few turns of a bandage. The proper application of the ice-bag is greatly facilitated by the attention of a skilful nurse. Suspension of the bag should be tried as a last resource. The length of time during which the application should be continued is to be decided by the condition of the patient, and the progress of the disease. Should the temperature, which must be taken every hour, fall below 100 deg., the bag must be removed, and replaced when it rises again to 102 deg. The extremities must be kept warm by hot flannels, or fomentations if necessary.

This remedy must be used with caution in the cases of aged, debilitated, or alcoholic subjects, and in such conditions Dr. Lees recommends the use of alcohol, and subcutaneous injections of strychnine during the application of the ice bag. The treatment of the pyrexia may now be considered apart from the use of the ice bag. The use of aconite for this purpose is entirely avoided at St. Mary's. Should the temperature not rise above 103·5, no active measures, beyond the occasional sponging of the patient, are taken to reduce it. A rise of temperature above 104 deg. is met by the administration of five grains of quinine, which may be repeated every four hours if necessary. Should the temperature in spite of these measures continue to rise, sponging with ice-cold water, or the use of the cold bath must be resorted to. Antipyrin and antifebrin are seldom used at St. Mary's for the purpose of reducing the temperature in pneumonia. During this stage of the disease it is customary to give some such mixture as the following every six hours: ℞. ammon. caul. gr. iv., pot. citrat. gr. xv., sp. ammon. co. ♏xx., sp. aeth. nit. ♏xxv., decoct. cinch co. ℥j. As regards alcohol it is seldom found necessary to prescribe it during this stage of the attack. A little may sometimes be given with advantage at the outset of the disease in aged or very young subjects, but ammonia and beef tea are almost equally efficacious in minimising the effects of any shock that may exist. In persons of intemperate habits, and in those cases in which the disease is of a severe type, alcohol may be required at an early stage of the disease. Sleeplessness may be treated with Dover's powder, given in ten grain doses at night Venesection is rarely employed at St. Mary's, but in those cases in which cyanosis is a marked feature, associated with a high tension pulse, the application of leeches over the right heart is frequently attended with very beneficial results.

During the stage of consolidation the treatment as above indicated may be continued, the heart is carefully watched, and the earliest symptoms of cardiac failure are anticipated by the use of alcohol, and digitalis added to the mixture. It is seldom found necessary to give more than twelve ounces of alcohol in the twenty-four hours, even in those cases in which delirium and a rapid pulse are marked features of the attack. In severe cases the digitalis is combined with strychnine and ether, and given every three hours while the dangerous symptoms continue. The bowels during this period may be kept open by means of salines. The occurrence of crisis is frequently associated with very considerable depression, and may call for the very free use of stimulants. Convalescence after pneumonia is usually rapid. During the early part of this period it is necessary to keep the bowels regular, and a more generous dietary may be gradually assumed. Alcohol, which, up to this point, has been administered in the form of brandy, should be gradually reduced in quantity, and is now most advantageously given in the form of port wine. Should the temperature remain normal, the patient may "get up" at the end of the first week of convalescence. A secondary rise of temperature, which is a fairly common occurrence during resolution, must be treated by means of quinine and tonics. The combination of a few grains of iodide of potassium with a bland preparation of iron, given with a bitter, is useful in hastening resolution. The use of counter irritants may also be employed for the same purpose. In weakly subjects, a generous diet and the administration of cod-liver oil are beneficial in promoting convalescence. Change of air is often necessary before a complete restoration to health is established, and it should be recommended in all cases.

In those cases in which recovery of the lung does not take place, it is of the first importance to maintain the patient's strength, and should the expectoration become fœtid, the use of antiseptic inhalations must be resorted to. If these measures fail to produce an amelioration of the symptoms, surgical interference may give relief.

